# Interest in the Endoscopic Endonasal Route as Access to the Parapharyngeal Space for Cervical Sympathetic Chain Schwannoma Resection: A Case Report

**DOI:** 10.7759/cureus.36461

**Published:** 2023-03-21

**Authors:** Felipe Gaia, José Renan Miranda Cavalcante-Filho, Marco Aurelio Franco de Godoy Belfort

**Affiliations:** 1 Neurosurgery, Hospital de Esperança, Presidente Prudente, BRA; 2 Otolaryngology - Head and Neck Surgery, Hospital de Esperança, Presidente Prudente, BRA

**Keywords:** neuro-oncology, neuroendoscopy, neurosurgery, endoscopic endonasal approach, cervical sympathetic chain schwannomas

## Abstract

Schwannomas are tumors originating from Schwann cells, about 45% of them are located in the head and neck region, including the parapharyngeal space (PFS). Vagus nerve schwannomas (VS) and cervical sympathetic chain schwannomas (CSCS) are most commonly found in this region. We present a case of CSCS located in the PFS of a middle-aged female patient who had surgery with the endoscopic endonasal approach (EEA). There have been no earlier reports of CSCS resection utilizing EEA, according to our literature search. To our knowledge, there is no scientific record of CSCS resection using EEA.

## Introduction

Schwannomas are tumors originating from Schwann cells, described by Verocay in 1908. Despite appearing in various parts of the body, about 45% of them are located in the head and neck region, including the parapharyngeal space (PPS). Vagus nerve schwannomas (VS) and cervical sympathetic chain schwannomas (CSCS) are most commonly found in this region [1].

The post-styloid compartment of PPS encases principal neurovascular structures, such as the cervical sympathetic chain; cranial nerves IX, X, XI, and XII, as well as the internal carotid artery (ICA), and internal jugular vein (IJV).

We present a case of CSCS located in the PPS of a middle-aged female patient who underwent surgery via the endoscopic endonasal approach (EEA). There are no reports in the literature on CSCS resection using EEA.

## Case presentation

Anamnesis and physical examination

A 43-year-old female patient was referred by the head and neck surgery team after a transoral biopsy of a palpable lesion in the right cervical region. The patient reported that for 18 months, a palpable and painless cervical mass was present on the right side of the neck with progressive growth. The patient denied having dysphonia, dysphagia, hoarseness, or any other symptoms associated with Horner syndrome (HS).

Neuroimaging

**Figure 1:**
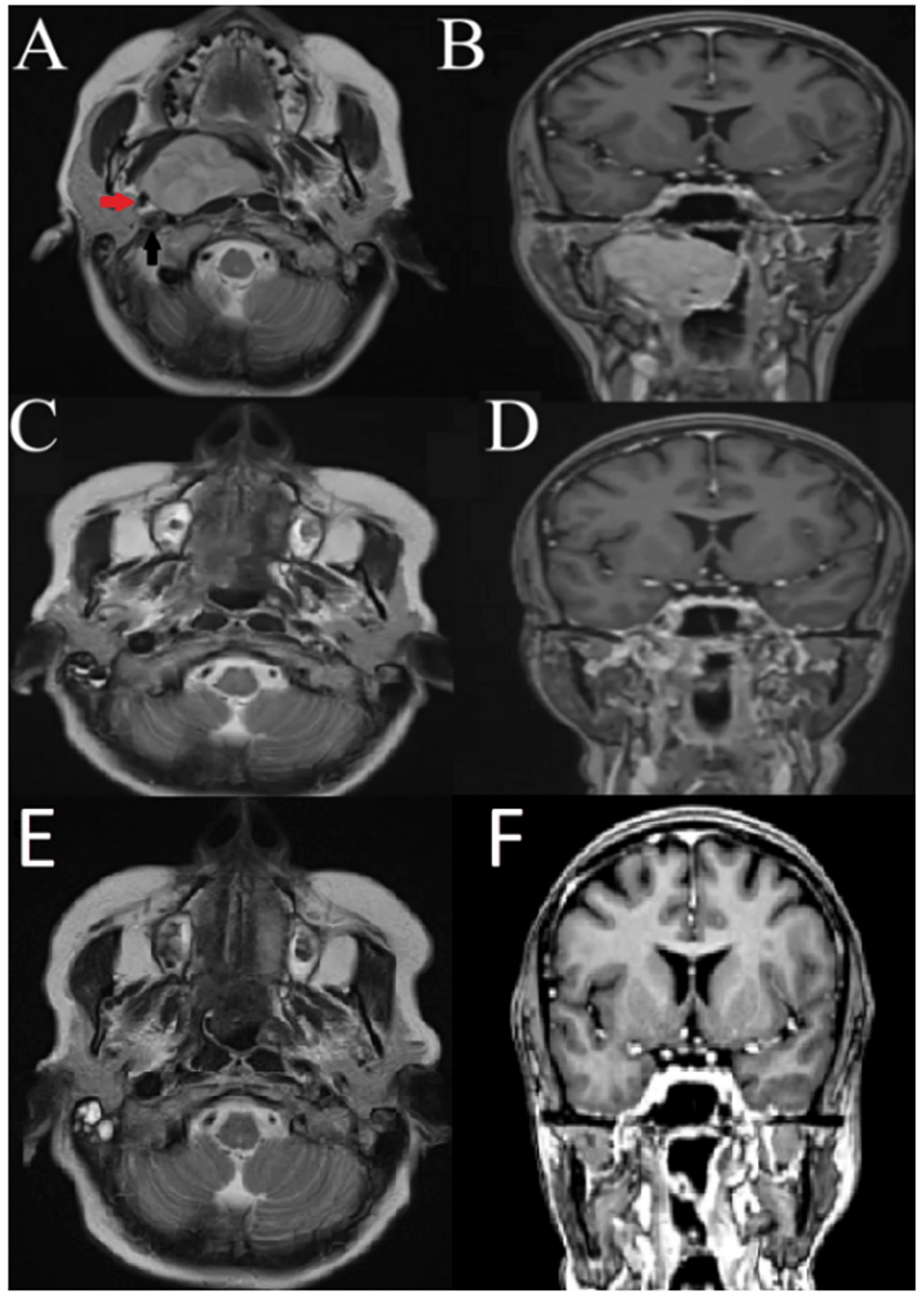
A: T2 axial MRI showing an expansive lesion in the right parapharyngeal space, laterally displacing the right common carotid artery (red arrow) and right internal jugular vein (black arrow). B: T1 with contrast coronal MRI showing an expansive lesion in the right parapharyngeal space. C, D: Axial and coronal MRI three months after lesion resection; E, F: Axial and coronal MRI 12 months after lesion resection.

A well-delimited expansive growth in the right parapharyngeal cavity, with homogeneous contrast uptake, estimated to be 5.6 cm, which extended to the rhino pharynx, was seen on the magnetic resonance imaging (MRI) of the skull. The lesion described displaced the IJV and ICA in the same posterolateral direction to the right (Figure 1).

Surgical technique

Given the upper parapharyngeal location, we decided to perform surgical resection through the EEA endonasal route with the aid of neuronavigation and ICA monitoring was performed with the aid of Doppler ultrasound.

**Figure 2:**
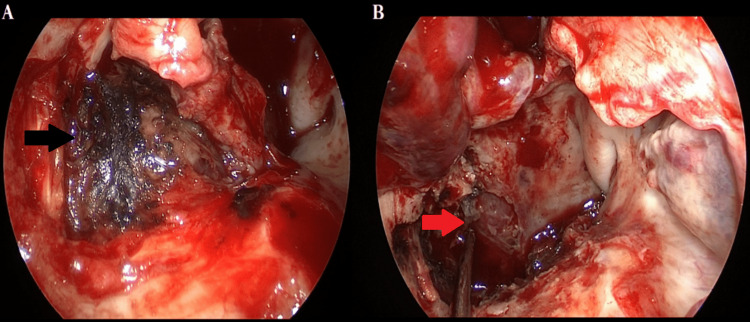
View of the endoscopic endonasal approach (EEA) approach. A: Yellowish lesion located in the right parapharyngeal space (PFS) with extension to the rhinopharynx after coagulation with bipolar (black arrow). B: Aspect after total resection with evidence at the bottom of the ipsilateral internal carotid artery (ICA) indicated by a microaspirator (red arrow), whose arrangement indicated the posterolateral limit of the lesion.

Initially, right turbinectomy was performed, followed by posterior septectomy, bilateral sphenoethmoidectomy, and finally increased antrostomy. The sphenopalatine foramen was identified, as well as the sphenopalatine and palatovaginal arteries, both of which were resected. Afterward, the resection of the posterolateral aspect of the maxillary sinus was performed which revealed the posterior outlook of the pterygopalatine fossa. The transpterygoid approach was performed by removing the inferior portion of the periosteum around the vidian nerve which allowed access to the base of the pterygoid processes. Extensive removal of the pterygoid process and medial and lateral pterygoid plates provided complete exposure of the upper portion of the PFS. In the right PFS, a yellowish fibroelastic lesion was identified whose posterolateral limits were closely related to the ipsilateral ICA. An ultrasonic aspirator was used to perform tumor debulking (Figure 2). Considering the size of the lesion, it was impossible to adequately identify the nerve of origin. After a review of hemostasis, a nasal mucosa flap, and nasal pack were inserted. The initial programming of the procedure would be the combination of EEA and cervicotomy with the aid of head and neck surgery. However, the EEA alone was able to resect the entire lesion. The procedure was performed without complications.

Postoperative evolution

The patient reported mild dysphagia on the first postoperative (POD), without any evidence of HS or first bite syndrome (FBS), and was discharged on the fourth POD.

**Figure 3:**
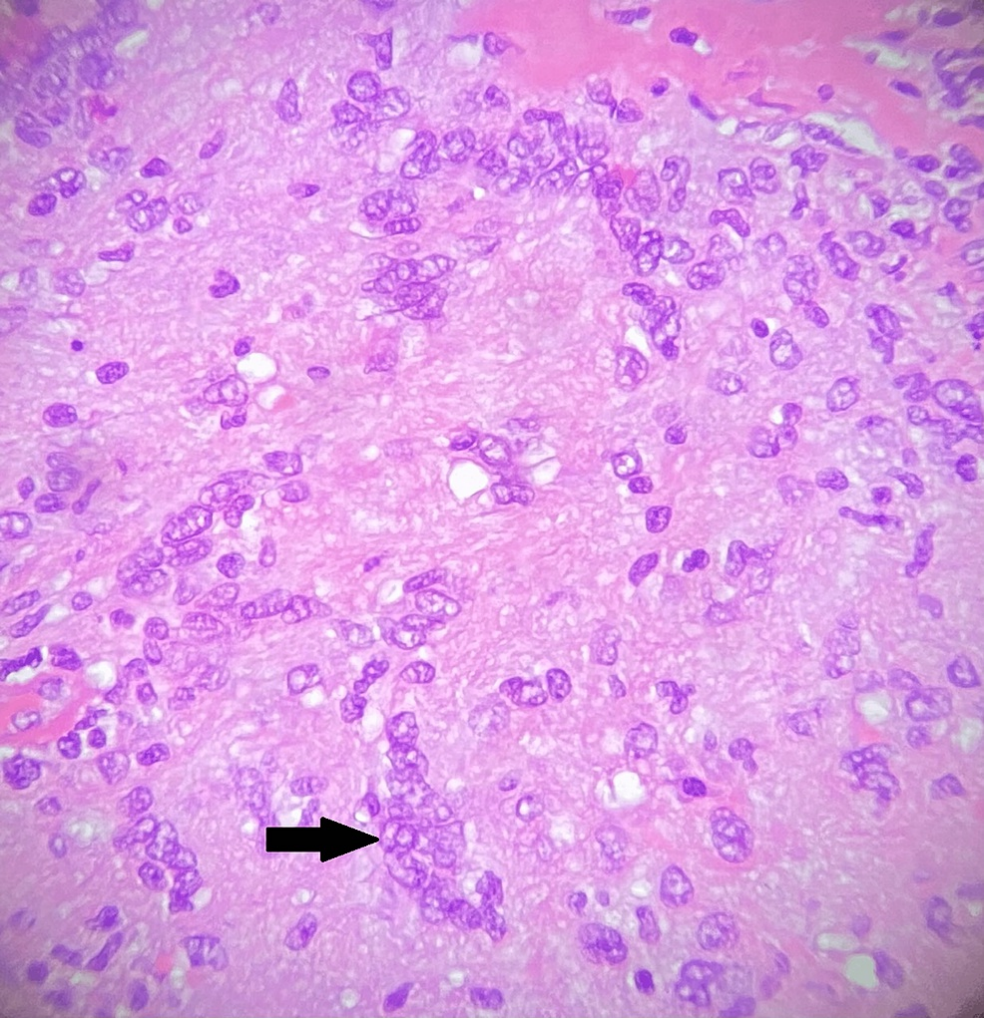
Microscopy image H&E stain at 400x showing Verocay bodies (black arrow).

The pathology examination of the lesion revealed that it was a schwannoma, and after three months and 12 months, the control MRI of the skull revealed a full excision of the lesion with no evidence of tumor recurrence. At the 12 months follow-up, the patient remained free of complaints or complications (Figure 3).

## Discussion

The vagus nerve is the most frequent origin of schwannoma found in the head and neck region. When large, they can cause compressive symptoms, such as pain and paresthesia, as well as dysphagia and hoarseness. Graffeo et al. [2[ compiled data from investigations which identified several patterns that can be used to differentiate these tumors from CSCS (Table 1). The following findings are considered in the diagnosis of CSCS: absence of separation by the tumor of the ICA and IJV, lateral displacement of the ICA, absence of HS and dysphagia and vocal cord paralysis, and homogeneous contrast enhancement. Such concepts, when applied in the case described, make it possible to diagnose CSCS with a reliability of about 99%, and a p-value of 0.001. [2]

**Table 1:** Analysis of schwannoma origin from clinical and radiological data Adapted from Graffeo et al. [2]. CSCS - cervical sympathetic chain schwannoma; ICA - internal carotid artery; IJV- internal jugular vein; VS - vagal schwannoma.

ICA/IJV Splaying	Medial ICA Displacement	Lateral ICA Displacement	Dysphagia/Vocal Cord Paralysis	Horner’s Syndrome	Peripheral Enhancement	Homogeneous Enhancement	VS	CSCS	p Value
Yes	Yes	-	-	-	-	-	86%	-	0,001
No	-	Yes	-	-	-	-	-	91%	0,006
Yes	Yes	Yes	Yes	-	-	-	90%	-	0,003
No	-	Yes	-	Yes	-	-	-	93%	0,002
Yes	Yes	-	-	-	Yes	-	97%	-	0,002
No	-	Yes	-	-	-	Yes	-	99%	0,001
Yes	Yes	-	Yes	-	Yes	-	100%	-	0,002
No	-	Yes	-	Yes	-	Yes	-	100%	0,002

A review that studied 1,143 PPS tumors showed that most of them are benign (82%). The most common subtype according to the aforementioned review is salivary gland tumors (45%), followed by schwannomas (41%). Surgical treatment is the conventional therapy for tumors located in the PFS, and it has as its main objective, the complete excision of the tumor while maintaining the surrounding noble structure [3]. FBS and transient HS are the most common complications in CSCS resection. FBS occurs due to an injury to the sympathetic chain, and it is characterized by radiating pain in the mandibular region that starts with the first bite and disappears with repeated chewing during meals [4].

In cases with PFS tumors, the transcervical approach (TCA) has historically been the most used route. TCA is advantageous because it allows direct access to the lower portion of the PFS, as well as a good visualization of the cranial nerves and control of the great vessels, reducing the risk of facial nerve injury and bleeding, respectively [3]. EEA has the advantage of decreasing the occurrences of cosmetic and functional complications associated with open surgery, as well as reducing hospital stay. The EEA can provide an adequate surgical window for the resection of PPS tumors, and it has the advantage of creating a direct line view of the internal structures, which allows easy identification of the PPS anatomy as the dissection progresses [5]. Its limitation is the access to the petrous portion of the ICA and the longer operative time when compared to open techniques [5]. The resection of lesions that are deep, large, and adherent to the ICA also becomes laborious for EEA, the latter being a possible contraindication for the endoscopic approach [5]. The most pervasive complication following open techniques was HS (91.1%) with a high prevalence of lesions > 4 cm. This condition can be transient or permanent and corroborates with a worse quality of life for patients [6]. The present case did not manifest HS, however, as there are no other cases treated exclusively with EEA in the literature, it is not possible to conclude the prevalence of this complication using the endoscopic technique.

## Conclusions

To our knowledge, there is no scientific record of CSCS resection using EEA. Understanding the endoscopic anatomy of the PFS can lead to improvements in morbidity associated with tumor resection in this dense neurovascular region, as well as reduce surgery time.
